# The double burden of malnutrition and environmental enteric dysfunction as potential factors affecting gut-derived melatonin in children under adverse environments

**DOI:** 10.3389/fnut.2023.1217173

**Published:** 2023-11-27

**Authors:** Alane N. Bezerra, Caroline L. Peixoto, Synara C. Lopes, Veralice M. S. Bruin, Pedro Felipe C. Bruin, Reinaldo B. Oriá

**Affiliations:** ^1^Laboratory of Tissue Healing, Ontogeny, and Nutrition, Department of Morphology, Faculty of Medicine, Federal University of Ceará, Fortalez, Brazil; ^2^Laboratory of Sleep and Biological Rhythms, Department of Clinical Medicine, Faculty of Medicine, Federal University of Ceará, Fortalez, Brazil

**Keywords:** melatonin, the double burden of malnutrition, environmental enteric dysfunction, intestinal microbiota, poverty, COVID-19

## Abstract

Poor environmental conditions combined with continuous unhealthy and unsafe diets may substantially increase the risk of a vicious cycle of enteric infections (EED–environmental enteric dysfunction) and malnutrition (DBM–double burden of malnutrition) in children. Gut melatonin, mainly produced by the intestinal microbiota, can modulate the composition, variety, and dynamics of the microbiota itself and may affect and be affected by intestinal microbiota alterations due to DBM and EED. 
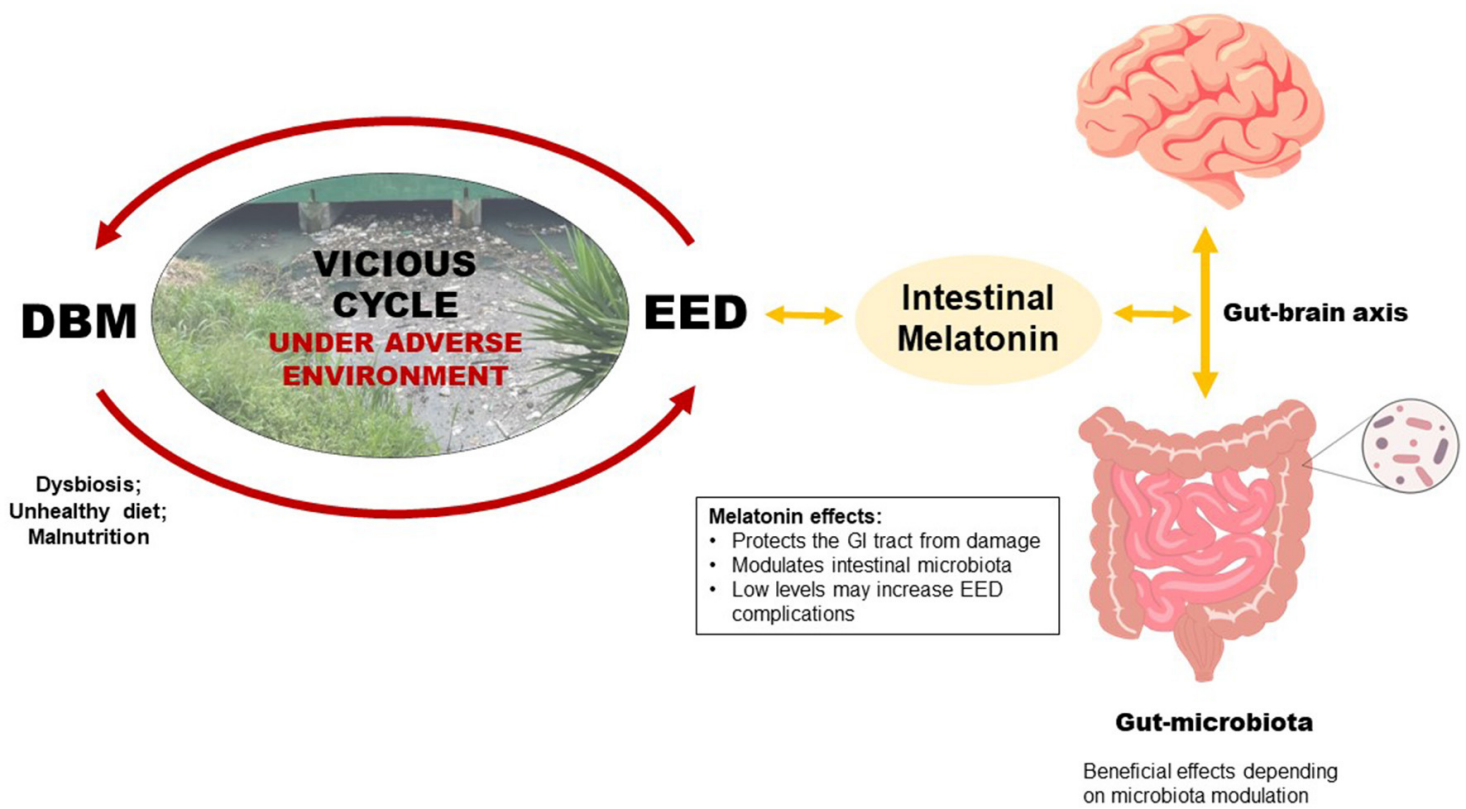

## Introduction

Proper nutrition is a cross-cutting component for ensuring a population's health and economic and social development. Due to accelerated nutritional, epidemiological, and demographic transition in certain regions of the developing world, especially in overpopulated urban areas, a double burden of malnutrition (DBM) has been a growing health concern, which may lead to important metabolic disorders during the lifespan with costly health care (1).

The double burden of malnutrition in children is increasing in developing countries and may occur in settings of poverty and inadequate sanitation (2). DBM is defined as a simultaneous occurrence of overweight/obesity and undernutrition afflicting countries at an individual or societal level, frequently associated with micronutrient deficiency (1). DBM may affect children living in developing countries in poverty conditions when a low-density nutrition intake is shifted to a high-caloric and high-fat Westernized diet, increasing the risk for non-communicable chronic diseases (2).

The COVID-19 pandemic may have fueled the prevalence of DBM in emerging economic countries, such as Brazil and India, which may lead to unprecedented and escalating increases in obesity rates (3). Under adverse environments and unhealthy diets, DBM may coexist with and favor environmental enteric dysfunction (EED), with underlying chronic intestinal inflammation and intestinal microbiota imbalances (4, 5).

Children from low-income families are often exposed to poor hygiene, unsanitary conditions, and difficult access to health care (6). When poor environmental conditions collide with continuous unhealthy and unsafe diets, such a combination may substantially increase the risk of a vicious cycle of enteric infections and malnutrition in children, disturbing their developmental trajectories (7). DBM and EED may be a cause and consequence of this vicious cycle, and if persistent, can lead to intestinal microbiota disturbances allowing more pathogenic microbial communities to thrive, with impaired intestinal barrier function and disrupted immune activation, with mucosal and systemic inflammatory effects (8).

Melatonin is a critical pineal gland-derived hormone regulating the circadian rhythm; nonetheless, it has been associated with immunoinflammatory functions in different body systems (9). Melatonin is also significantly produced by the gastrointestinal (GI) tract, which harbors highly expressed melatonin receptors, and regulates the intestinal barrier function (10). This opinion paper brings to attention that DBM compounded with the EED in growing children under adverse environments may negatively influence the intestinal microbiota homeostasis and hence the GI tract-related melatonin function.

## Gut derived-melatonin

Melatonin, N-acetyl-5-methoxy tryptamine, is a tryptophan-derived hormone synthesized mainly by the pineal gland but also by the retina, platelets, skin, and intestinal mucosa (9, 11). In the GI tract, melatonin is produced by enteroendocrine, endothelial, natural killer cells, and intestinal bacteria (10). In Wistar rats, gut melatonin levels are markedly high, reaching about 4–100 ng/g of wet organ weight (12). Intestinal melatonin is produced even during daylight hours when its synthesis by the pineal gland is low. Notably, animals lacking the pineal gland show stable amounts of melatonin in the GI tract (13).

Melatonin receptors (MT) are widely distributed at various sites within the Gl tract, including the intestinal mucosa. MT1 and MT2 receptors are found in blood vessels, epithelium, submucosa, and myenteric plexus. In the large intestine, MT1 and MT2 are more expressed in the epithelium (14). In addition, the enzymes necessary for melatonin synthesis are highly expressed in the GI tract (15). When intestinal inflammation prevails, changes in gene expression can lead to lower amounts of melatonin (14).

The rat intestinal mucosa undergoes morphological changes, with increased inflammatory responses, when endogenous melatonin suppression occurs following acute inhibition of MT1 and MT2 receptors by luzindole (16). Conversely, an association between bacteria that produce short-chain fatty acids (*Alistipes sp* and *Blautia sp*) with increased expression of melatonin has been found in the colon (17). Melatonin supplementation seems to have a protective action on the intestinal mucosa, improving pathogenic microbial composition in the colon, helping to prevent or treat intestinal infections (18).

Several factors, including diet and intestinal microbiota, influence intestinal melatonin levels. In absolute values, the amount of gut melatonin is 400 times higher than the pineal gland. Reductions in endogenous melatonin affect the intestinal microbiota and intriguingly trigger Alzheimer's disease-like phenotypes, including hippocampal Iba-1 activation, Aβ protein deposition, with impaired spatial memory ability in mice (11). Another source of intestinal melatonin is the intestinal microbiota, which can also induce colonic melatonin receptor expression by a mechanism of action involving short-chain fatty acids (17). The exogenous use of melatonin causes changes in the intestinal microbiota, which help the melatonergic system's function with increased intestinal epithelial regeneration (19).

Melatonin exogenous administration improves already installed intestinal damage, such as mucosal disruption and neutrophil infiltration, favoring antioxidative processes, reducing the generation of oxygen free radicals, and protecting the integrity of intestinal mucosal cells (20). Melatonin supplementation influences appetite, improving satiety and affecting plasma leptin levels, which are higher in supplemented individuals (21), suggesting a role for melatonin in regulating food intake.

Melatonin protects the intestinal barrier function, mainly due to its anti-inflammatory and antioxidant actions, and increases the abundance of bacterial populations (22). The gut microbiota is important in modulating the metabolism of tryptophan, an essential amino acid precursor to melatonin. Tryptophan metabolization pathways also exist in some members of the human intestinal microbiota, such as *Clostridium sporogenes* and *Ruminococcus gnavus*, which can decarboxylate tryptophan into the neurotransmitter tryptamine in the large intestine (23).

## Gut-derived melatonin may be affected by intestinal microbiota alterations due to the double burden of malnutrition and EED

Some factors can interfere with the gut microbiome, such as diet, genetics, age, gender, lifestyle, infections, diseases, and exposure to maternal and environmental microbiota (24). Genetics can explain changes in this microbiota by up to 12%. The dietary pattern modifies the microbiota's composition, changing the proportion between the phyla and the variety of microorganisms and explaining this variation by up to 57% (25). The gut microbiota is important in the gut-brain axis as it regulates the secretion of brain hormones, such as brain-gut peptides from intestinal endocrine cells, and bacterial compounds can cross the blood-brain barrier, regulating brain functions (26). We do not know whether altered microbiota and endogenous intestinal melatonin crosstalk to affect brain functions in children. This is an important gap in knowledge that should be addressed by innovative research.

Microbiota imbalance toward reductions in commensal bacteria, with alterations in the composition and quantity of intestinal microorganisms, is a key factor affecting gut nutrient bioavailability (24). Intestinal microbiota dysregulation facilitates and is facilitated by the luminal-to-blood translocation of pathogenic bacteria, with adverse effects on the intestinal epithelial barrier homeostasis, compromising its modulation by commensal bacteria (27).

A dietary pattern characterized by a high-fat content induces lipogenesis and causes intestinal microbiota imbalance. Oral melatonin supplementation in mice challenged with high fat intake leads to a greater diversity of the intestinal microbiome, characterized by a relative abundance of *Bacteroides, Alistipes*, and *Parasutterella* and reduced numbers of *Lactobacilli*. Notably, melatonin effects on the intestinal microbiota were reversed in animals treated with antibiotics (28). Melatonin supplementation alters the intestinal microbiota constitution, reduces the Firmicutes against Bacteroidetes, increases *Akkermansia*, and adjusts the abundance of *Alistipes, Anaerotruncus*, and *Desulfovibrionaceae* to previous levels, with beneficial effects against obesity, insulin resistance, hepatic steatosis, and low-grade inflammation (29). The impact of antibiotics use and melatonin supplementation (4 mg/kg in drinking water for 2 weeks) on high-fat diet-induced intestinal inflammation and gut dysbiosis has been investigated in rats. The findings reveal that even a brief exposure to a high-fat diet leads to a state of hepato-intestinal inflammation and shifts in bacterial populations that can be exacerbated through antibiotic administration but ameliorated by melatonin supplementation (30). Melatonin signaling may be a communication link between the intestine and the central nervous system, as it modulates the circadian rhythm, intestinal microbial metabolism, and intestinal immune system, activating the release of cytokines (10).

Children afflicted with EED often live in poor settings of the developing world, especially in tropical areas with relatively yearly constant daylight, thus affecting circulating melatonin levels (31). Lifestyle habits, high-caloric Western diets, and other factors influence melatonin synthesis and intestinal inflammation (28). High-stress levels can impact the pineal production and release of melatonin. The characteristics of ambient light also affect this production and directly impact physiological and immune functions (32).

Data on melatonin levels and intestinal barrier function biomarkers are still scarce in the literature, and such a paucity of studies with EED experimental models hamper findings from being applied in clinical settings. In addition to its antioxidant function, melatonin may contribute to increasing mucosal blood flow, strengthening the GI and immune system, controlling fecal moisture, reducing intestinal peristalsis, prolonging intestinal transit time, and protecting the GI tract from damage caused by digestive enzymes and hydrochloric acid, altering intestinal secretions (22). This favors epithelial regeneration and increases local microcirculation, promoting lower intestinal permeability.

A gut microbial community with a reduced relative abundance of *Bacteroides* and increased *Lactobacillus* and *Firmicutes* was found to be associated with marked intestinal permeability and systemic and local inflammation in an endogenous melatonin reduction mouse model (33). In addition, there was less resistance to stress when subjected to high-fat consumption, influencing weight gain and the development of hepatic steatosis. Fecal microbiota transplantation improves systemic inflammation and intestinal permeability by modulating the gut microbiota.

A healthy intestinal microbiota and reduced circulating LPS/endotoxemia would facilitate melatonin-protective antioxidant functions and improve chronic inflammation (24). Of note, maternal melatonin supplementation had a significant effect on the intestinal microbiota and decreased inflammatory mediators in the offspring following LPS injection (34). Accumulating evidence supports endogenous melatonin's influence on the intestinal microbiome, homeostasis, and stress resistance (33), suggesting that its reduction is a risk factor for EED complications.

The gut microbiome can directly influence children's growth. In a model of chronic malnutrition induced by diet, without intestinal inflammation, the mouse microbiota enriched by *Lactiplantibacillus plantarum* (strain LpWJL) provided greater growth and metabolic and hormonal alterations, with higher levels of IGF-1 and insulin. This bacterium promotes the signaling of NOD2, an innate immunological receptor in the crypts that is inhibited due to malnutrition, with improvements in intestinal cell proliferation and nutritional absorption, increasing mouse growth (35). Melatonin found in the breastmilk can influence the composition, variety, and dynamics of the intestinal microbiota over time, as well as modulating absorption of molecules by the intestinal epithelia (36). This effect may regulate the intestinal microbiota and influence the short and long-term malnutrition states.

Intestinal pathogenic microbial populations may impair the beneficial effects of melatonin. Melatonin supplementation to mice challenged by a colitis model led to increased intestinal inflammation and permeability with augmented tissue levels of TNF and circulating mononuclear cells and neutrophils. The pro-inflammatory effect of melatonin was associated with reduced *Bacteroidetes* and abundance in the *Actinobacteria* and *Verrucomicrobia* phyla, and when the dysbiosis was corrected, this effect was not observed (37).

As far as we know the scientific literature on melatonin and EED/DBM is still missing, therefore it is difficult to distinct the underlying effects and mechanisms of melatonin's efficacy in such conditions (EED/DBM) comparing to other well-recognized gastrointestinal diseases. Up to date, beneficial effects of melatonin supplementation has been found in animal models of obesity and metabolic syndrome (38), intestinal bowel disease (39) and irritable bowel syndrome (40), mostly by antioxidant, anti-inflammatory and regulatory intestinal microbiota's effects. We expect that some of the underlying mechanisms of melatonin's protective mechanisms on these conditions also happen to EED/DBM.

One gap of knowledge is that most of the melatonin studies come from experimental models and more clinical studies are needed to address the effects of melatonin on the double burden of malnutrition, especially in children under adverse environments.

## Conclusion

This opinion article raises awareness that GI-tract-related melatonin function may be altered by DBM and EED (both conditions may interfere with intestinal microbiota), negatively affecting children living in adverse environments. More studies are needed to assess further the gut microbiome's modulatory effects under DBM and EED, and their crosstalk with melatonin function. Improvements in this knowledge may favor breakthrough nutritional interventions to ameliorate nutrient deficiency and healthier intestinal microbiota to halt short and late-onset overweight/obesity and its long-term risks. Further research is warranted to address whether melatonin supplementation can help to improve pathogenic gut microbiota and intestinal inflammation in experimental models of DBM and EED, possibly guiding future clinical studies in pediatric populations.

## Author contributions

All authors listed have made a substantial, direct, and intellectual contribution to the work and approved it for publication.

## References

[1] Wells JC Sawaya AL Wibaek R Mwangome M Poullas MS Yajnik CS. The double burden of malnutrition: aetiological pathways and consequences for health. Lancet. (2020) 395:75–88. 10.1016/S0140-6736(19)32472-9 31852605 PMC7613491

[2] Leocádio PCL Lopes SC Dias RP Alvarez-Leite JI Guerrant RL Malva JO. The transition from undernutrition to overnutrition under adverse environments and poverty: the risk for chronic diseases. Front Nutr. (2021) 8:1–5. 10.3389/fnut.2021.676044 33968973 PMC8102690

[3] Littlejohn P Finlay BB. When a pandemic and an epidemic collide: COVID-19, gut microbiota, and the double burden of malnutrition. BMC Med. (2021) 19:1–8. 10.1186/s12916-021-01910-z 33504332 PMC7840385

[4] Oriá RB Empadinhas N Malva JO. Editorial: Interplay between nutrition, the intestinal microbiota and the immune system. Front Immunol. (2020) 11:1758. 10.3389/fimmu.2020.01758 32849628 PMC7423988

[5] Pai SR Kurpad A V Kuriyan R Mukhopadhyay A. Intraindividual double burden of malnutrition: the contribution of the infant gut microbiome. Asia Pac J Clin Nutr. (2022) 31:157–66. 10.6133/apjcn.202206_31(2).0001 35766551

[6] Lima AAM Oriá RB Soares AM Filho JQ De Sousa F Abreu CB. Geography, population, demography, socioeconomic, anthropometry, and environmental status in the MAL-ED cohort and case-control study sites in Fortaleza, Ceará, Brazil. Clin Infect Dis. (2014) 59:S287–94. 10.1093/cid/ciu438 25305299

[7] Oriá RB Murray-Kolb LE Scharf RJ Pendergast LL Lang DR Kolling GL. Early-life enteric infections: Relation between chronic systemic inflammation and poor cognition in children. Nutr Rev. (2016) 74:374–86. 10.1093/nutrit/nuw008 27142301 PMC4892302

[8] McCormick BJJ Murray-Kolb LE Lee GO Schulze KJ Ross AC Bauck A. Intestinal permeability and inflammation mediate the association between nutrient density of complementary foods and biochemical measures of micronutrient status in young children: Results from the MAL-ED study. Am J Clin Nutr. (2019) 110:1015–25. 10.1093/ajcn/nqz151 31565748 PMC6766446

[9] Pham L Baiocchi L Kennedy L Sato K Meadows V Meng F. The interplay between mast cells, pineal gland, and circadian rhythm: Links between histamine, melatonin, and inflammatory mediators. J Pineal Res. (2021) 70:12699. 10.1111/jpi.12699 33020940 PMC9275476

[10] Ma N Zhang J Reiter RJ Ma X. Melatonin mediates mucosal immune cells, microbial metabolism, and rhythm crosstalk: a therapeutic target to reduce intestinal inflammation. Med Res Rev. (2020) 40:606–32. 10.1002/med.21628 31420885

[11] Zhang B Chen T Cao M Yuan C Reiter RJ Zhao Z. Gut microbiota dysbiosis induced by decreasing endogenous melatonin mediates the pathogenesis of alzheimer's disease and obesity. Front Immunol. (2022) 13:1–13. 10.3389/fimmu.2022.900132 35619714 PMC9127079

[12] Huether G. Melatonin synthesis in the gastrointestinal tract and the impact of nutritional factors on circulating melatonin. Ann N Y Acad Sci. (1994) 719:146–58. 10.1111/j.1749-6632.1994.tb56826.x 8010590

[13] Bubenik GA Brown GM. Pinealectomy reduces melatonin levels in the serum but not in the gastrointestinal tract of rats. Biol Signals. (1997) 6:40–4. 10.1159/000109107 9098522

[14] Söderquist F Hellström PM Cunningham JL. Human gastroenteropancreatic expression of melatonin and its receptors MT1 and MT2. PLoS ONE. (2015) 10:1–18. 10.1371/journal.pone.0120195 25822611 PMC4378860

[15] Paulose JK Cassone C V Cassone VM. Aging, melatonin biosynthesis, and circadian clockworks in the gastrointestinal system of the laboratory mouse. Physiol Genomics. (2019) 51:1–9. 10.1152/physiolgenomics.00095.2018 30444453 PMC6383550

[16] Matos RS Oriá RB Bruin PFC Pinto D V Viana AFSC Santos FA. Acute blockade of endogenous melatonin by luzindole, with or without peripheral LPS injection, induces jejunal inflammation and morphological alterations in Swiss mice. Brazilian J Med Biol Res. (2021) 54:1–10. 10.1590/1414-431X2021E11215 34431873 PMC8389610

[17] Wang B Zhang L Zhu SW Zhang JD Duan LP. Short chain fatty acids contribute to gut microbiota-induced promotion of colonic melatonin receptor expression. J Biol Regul Homeost Agents. (2019) 33:763–71. 31204469

[18] Gao T Wang Z Dong Y Cao J Lin R Wang X. Role of melatonin in sleep deprivation-induced intestinal barrier dysfunction in mice. J Pineal Res. (2019) 67:1–16. 10.1111/jpi.12574 30929267

[19] Lin R Wang Z Cao J Gao T Dong Y Chen Y. Role of melatonin in murine “restraint stress”-induced dysfunction of colonic microbiota. J Microbiol. (2021) 59:500–12. 10.1007/s12275-021-0305-7 33630247

[20] Ercan F Çetinel S Contuk G Çikler E Sener G. Role of melatonin in reducing water avoidance stress-induced degeneration of the gastrointestinal mucosa. J Pineal Res. (2004) 37:113–21. 10.1111/j.1600-079X.2004.00143.x 15298670

[21] Albreiki MS Shamlan GH BaHammam AS Alruwaili NW Middleton B Hampton SM. Acute impact of light at night and exogenous melatonin on subjective appetite and plasma leptin. Front Nutr. (2022) 9:1–10. 10.3389/fnut.2022.1079453 36562040 PMC9763572

[22] Gong YQ Hou FT Xiang CL Li CL Hu GH Chen CW. The mechanisms and roles of melatonin in gastrointestinal cancer. Front Oncol. (2022) 12:1–10. 10.3389/fonc.2022.1066698 36591447 PMC9798083

[23] Williams BB Van Benschoten AH Cimermancic P Donia MS Zimmermann M Taketani M. Discovery and characterization of gut microbiota decarboxylases that can produce the neurotransmitter tryptamine. Cell Host Microbe. (2014) 16:495–503. 10.1016/j.chom.2014.09.001 25263219 PMC4260654

[24] Kho ZY Lal SK. The human gut microbiome - a potential controller of wellness and disease. Front Microbiol. (2018) 9:1835. 10.3389/fmicb.2018.01835 30154767 PMC6102370

[25] Zhang C Zhang M Wang S Han R Cao Y Hua W. Interactions between gut microbiota, host genetics and diet relevant to development of metabolic syndromes in mice. ISME J. (2010) 4:232–41. 10.1038/ismej.2009.112 19865183

[26] Pan S Guo Y Hong F Xu P Zhai Y. Therapeutic potential of melatonin in colorectal cancer: Focus on lipid metabolism and gut microbiota. Biochim Biophys Acta - Mol Basis Dis. (2022) 1868:166281. 10.1016/j.bbadis.2021.166281 34610472

[27] Iesanu MI Denise C Zahiu M Dogaru I Chitimus DM Pircalabioru GG. Melatonin - microbiome two-sided interaction in dysbiosis-associated conditions. Antioxidants (2022) 11:2244. 10.3390/antiox11112244 36421432 PMC9686962

[28] Yin J Li Y Han H Chen S Gao J Liu G. Melatonin reprogramming of gut microbiota improves lipid dysmetabolism in high-fat diet-fed mice. J Pineal Res. (2018) 65:0–2. 10.1111/jpi.12524 30230594

[29] Xu P Wang J Hong F Wang S Jin X Xue T Jia L Zhai Y. Melatonin prevents obesity through modulation of gut microbiota in mice. J Pineal Res. (2017) 62:12399. 10.1111/jpi.12399 28199741

[30] Yildirim A Arabacl Tamer S Sahin D Bagriacik F Kahraman MM Onur ND. The effects of antibiotics and melatonin on hepato-intestinal inflammation and gut microbial dysbiosis induced by a short-term high-fat diet consumption in rats. Br J Nutr. (2019) 122:841–55. 10.1017/S0007114519001466 31217044

[31] Pazarci P Kaplan H Alptekin D Yilmaz M Lüleyap U Singirik E. The effects of daylight exposure on melatonin levels, Kiss1 expression, and melanoma formation in mice. Croat Med J. (2020) 61:55–61. 10.3325/cmj.2020.61.55 32118379 PMC7063558

[32] Baekelandt S Mandiki SNM Kestemont P. Are cortisol and melatonin involved in the immune modulation by the light environment in pike perch Sander lucioperca? J Pineal Res. (2019) 67:1–12. 10.1111/jpi.12573 30924977

[33] Zhang Y Lang R Guo S Luo X Li H Liu C. Intestinal microbiota and melatonin in the treatment of secondary injury and complications after spinal cord injury. Front Neurosci. (2022) 16:1–15. 10.3389/fnins.2022.981772 36440294 PMC9682189

[34] Li F Lai J Ma F Cai Y Li S Feng Z. Maternal melatonin supplementation shapes gut microbiota and protects against inflammation in early life. Int Immunopharmacol. (2023) 120:110359. 10.1016/j.intimp.2023.110359 37257272

[35] Schwarzer M Gautam UK Makki K Lambert A Brabec T Joly A. Microbe-mediated intestinal NOD2 stimulation improves linear growth of undernourished infant mice. Science. (2023) 379:826–33. 10.1126/science.ade9767 36821686

[36] Gombert M Codoñer-Franch P. Melatonin in early nutrition: long-term effects on cardiovascular system. Int J Mol Sci. (2021) 22:6809. 10.3390/ijms22136809 34202781 PMC8269134

[37] da Silva JL Barbosa LV Pinzan CF Nardini V Brigo IS Sebastião CA. The microbiota-dependent worsening effects of melatonin on gut inflammation. Microorganisms. (2023) 11:460. 10.3390/microorganisms11020460 36838425 PMC9962441

[38] Cano Barquilla P Pagano ES Jiménez-Ortega V Fernández-Mateos P Esquifino AI Cardinali DP. Melatonin normalizes clinical and biochemical parameters of mild inflammation in diet-induced metabolic syndrome in rats. J Pineal Res. (2014) 57:280–90. 10.1111/jpi.12168 25113124

[39] Vaghari-Tabari M Moein S Alipourian A Qujeq D Malakoti F Alemi F. Melatonin and inflammatory bowel disease: From basic mechanisms to clinical application. Biochimie. (2023) 209:20–36. 10.1016/j.biochi.2022.12.007 36535545

[40] Lv MD Wei YX Chen JP Cao MY Wang QL Hu S. Melatonin attenuated chronic visceral pain by reducing Nav18 expression and nociceptive neuronal sensitization. Mol Pain. (2023) 19:1–15. 10.1177/17448069231170072 37002193 PMC10123881

